# Long-term monitoring in a microfluidic system to study tumour spheroid response to chronic and cycling hypoxia

**DOI:** 10.1038/s41598-019-54001-8

**Published:** 2019-11-28

**Authors:** Samantha M. Grist, S. Soroush Nasseri, Loïc Laplatine, Jonathan C. Schmok, Dickson Yao, Jessica Hua, Lukas Chrostowski, Karen C. Cheung

**Affiliations:** Department of Electrical and Computer Engineering, The University of British Columbia, Vancouver, Canada

**Keywords:** Biomedical engineering, Assay systems

## Abstract

We demonstrate the application of a microfluidic platform combining spatiotemporal oxygen control and long-term microscopy monitoring to observe tumour spheroid response to hypoxia. The platform is capable of recreating physiologically-relevant low and cycling oxygen levels not attainable in traditional cell culture environments, while image-based monitoring visualizes cell response to these physiologically-relevant conditions. Monitoring spheroid cultures during hypoxic exposure allows us to observe, for the first time, that spheroids swell and shrink in response to time-varying oxygen profiles switching between 0% and 10% O_2_; this swelling-shrinkage behaviour appears to be driven by swelling of individual cells within the spheroids. We also apply the system to monitoring tumour models during anticancer treatment under varying oxygen conditions. We observe higher uptake of the anticancer agent doxorubicin under a cycling hypoxia profile than under either chronic hypoxia or *in vitro* normoxia, and the two-photon microscopy monitoring facilitated by our system also allows us to observe heterogeneity in doxorubicin uptake within spheroids at the single-cell level. Combining optical sectioning microscopy with precise spatiotemporal oxygen control and 3D culture opens the door for a wide range of future studies on microenvironmental mechanisms driving cancer progression and resistance to anticancer therapy. These types of studies could facilitate future improvements in cancer diagnostics and treatment.

## Introduction

Cell-based drug screening methods are used early in the drug development process to identify potential cancer treatments by studying effects such as viability or changes in cell phenotype in response to a potential treatment^1^. Traditional *in vitro* screening of cancer treatments uses cells that are grown in two-dimensional (2D) monolayers in environments like petri dishes. Many factors (including cell-cell and cell-extracellular matrix signalling) influencing cell behaviour are not reproduced in the 2D monolayer cell cultures commonly employed in traditional screening assays, and it is thought that this poor reproduction of the *in vivo* microenvironment contributes to the high attrition rate of cancer drugs in clinical trials (80%^2^ to 95%^3^ of the cancer treatment candidates that enter phase I clinical trials never make it to market authorization). In contrast, three-dimensional (3D) cell cultures have the potential to better replicate *in vivo* responses as they can reproduce cell-cell and cell-matrix interactions as well as diffusion gradients of drugs, nutrients, oxygen, and pH^4–7^, and dynamic changes in microenvironmental parameters such as stiffness^8^.

Tissue hypoxia (or inadequate oxygen level) in tumours is one aspect of the microenvironment known to contribute to resistance to radiation therapy and anticancer drugs^9^. Hypoxia is often present within solid tumours because the fast proliferation of tumour cells leads to abnormal vasculature, where cells may be located too far from blood vessels to receive adequate oxygen supply^10,11^. Cancer can also result in issues such as anemia that lead to a reduced ability of blood to carry oxygen^12^. In addition to the requirement of molecular oxygen itself for certain therapies such as antibiotics that induce DNA strand breaks^13^, a number of adaptations can be made by tumour cells upon exposure to hypoxic environments; these adaptations can confer either resistance or sensitivity to different chemotherapies. The mechanisms by which hypoxic cells develop resistance and sensitivity to chemotherapeutic agents have been studied and reviewed in several publications^13–16^.

There are a variety of regimes and subtypes of hypoxia that induce changes in tumour cells. Although hypoxia is often defined as oxygen levels below 8–10 mm Hg (1.05–1.32% of 1 atm), levels below 25–30 mm Hg (3.29–3.95%) have been shown to significantly reduce the efficacy of treatment by X- and γ-radiation as well as certain immunotherapies^12^. Differences in the hypoxic oxygen levels between 0%, 0.1%, 1%, and 5% can result in different cell responses due to the differences in Hypoxia Inducible Factor (HIF) activation, metabolism, and proteomic and genomic changes between these oxygen conditions^17^. The importance of the degree and duration of hypoxia on cellular changes suggests that a high degree of control over the oxygen environment is essential in studying these effects *in vitro*; this control has been historically lacking from *in vitro* cell culture environments as well as environments for drug screening.

In addition to chronic hypoxia, it is thought that the irregular blood vessels in tumours can be intermittently perfused due to vascular remodelling as well as endothelial cell contractions closing and opening blood vessels^18^. This intermittent perfusion leads to transient cycles of intermittent or cycling hypoxia^19–23^ that can promote tumour aggressiveness^24^ and a metastatic phenotype in breast cancer^25^. Cycling hypoxia is also implicated in resistance to treatment: it elicits a robust HIF-1 response and is linked with glioblastoma chemoresistance as well as tumour and stromal resistance to radiation therapy via tumour cell secretion of pro-survival factors^26–28^. Large temporal fluctuations in blood flow and oxygenation within tumours have been observed in animal tumour models^21,29,30^ and human tumours^31–33^, with time scales ranging from several cycles per hour to cycles in days^23,34^. A major limitation of traditional cell culture environments such as stationary well plates is that even with hypoxia control chambers and incubators, they can only reproduce cycles on the order of hours because of the large distances through which oxygen needs to diffuse through media to reach the cells^35^. Another drawback to traditional culture environments is their use of plastics such as polystyrene, which can sequester oxygen within the polymer matrix for long periods of time because of the polymer’s oxygen solubility combined with its relatively low diffusion coefficient; this oxygen sequestration prevents the recreation of physiologically-relevant very low oxygen levels^36^.

Microfluidic systems are a promising platform to mitigate these challenges for the study of cycling hypoxia due to the precise oxygen control facilitated by their small size scales^37^. Spatiotemporal oxygen control within microfluidic systems, reproducing oxygen cycling times of up to 1–10 minutes/cycle, has been demonstrated for a number of applications including studying pancreatic islet cells^38^, brain slices^39^, cardiomyocytes^40^, red blood cell velocity^41^, endothelial cells^42,43^, and tumour cells in 3D culture^44^. We have developed a microfluidic system using a similar hydration layer to that developed by Wood, *et al*. for the study of red blood cell velocity^41^, optimized for long-term (multi-day) monitoring of 3D cell cultures under a high degree of oxygen control^45^. Using integrated optical oxygen sensors within the cell culture channel, we demonstrated oxygen switching times of less than 10 minutes, as well as a precise degree of oxygen control (down to 0.05% of 1 atm) alongside homogeneous oxygen levels and multi-day cycling profiles within the cell culture channel^45^.

In this work, we demonstrate the utility of our microfluidic platform for studying 3D cell cultures within a controlled environment. Our platform facilitates precise spatiotemporal oxygen control over 3D cultures, alongside the ability to dynamically monitor cell behaviour using transmitted light, fluorescence, and optical sectioning microscopy. The combination of precise, cyclic oxygen control with monitoring of cell culture response allows us to demonstrate for the first time that tumour spheroids can dynamically swell and shrink when exposed to cycling oxygen profiles, and that this swelling appears to be driven by swelling of the individual tumour cells. We also show how the platform can be used for the screening of cancer treatments under controllable hypoxic conditions. For the first time, we demonstrate a cycling hypoxia dependence in spheroids’ accumulation of doxorubicin and size behaviour during treatment by integrating precise microfluidic oxygen control, 3D cell culture, and long-term two-photon microscopy monitoring during the 72 h drug treatment.

## Results

### MCF-7 breast tumour spheroids swell under 0% oxygen and subsequently shrink under 3% and 10% oxygen

**Figure 1:**
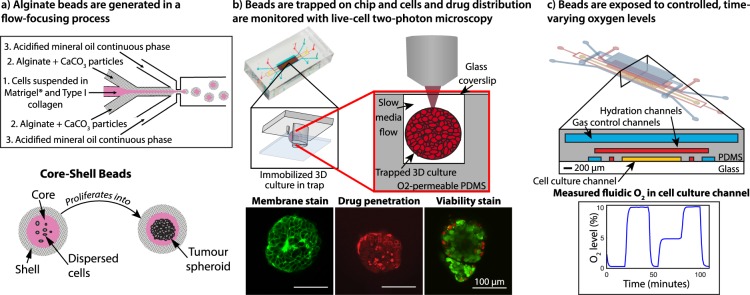
Concept of the microfluidic system for tumour spheroid generation, culture, oxygen control, and microscopy monitoring. (**a**) Disperse tumour cells form tumour spheroids within microfluidically-generated hydrogel droplets that gel to form microbeads. (**b**) After proliferation into tumour spheroids, the beads are trapped on-chip and monitored with two-photon microscopy. (**c**) A microfluidic oxygen control system facilitates the creation of controlled time-varying oxygen environments around the tumour spheroids, while monitoring permits the study of the effects of these oxygen environments.

We used our microfluidic system^45^ (concept depicted in Fig. 1) to study the response of tumour spheroids cultured in alginate hydrogel core-shell beads containing collagen I and Matrigel®. Hydrogel beads were generated in a flow-focusing process, which entrapped disperse breast tumour cells (MCF-7) within the bead core. After ~14 days, the cells proliferate to form tumour spheroids, as described in our previous work^46,47^. MCF-7 spheroid-containing beads from two different batches were trapped on-chip various times (14 days, 21 days, 30 days) after bead formation. Spheroids were exposed on-chip to cycling oxygen profiles of varying duration: gases were cycled between 0%, 3%, and 10% oxygen (representing severe hypoxia, typical tumour oxygenation, and physiologic normoxia, respectively). All mixed gases contained 5% CO_2_.

**Figure 2:**
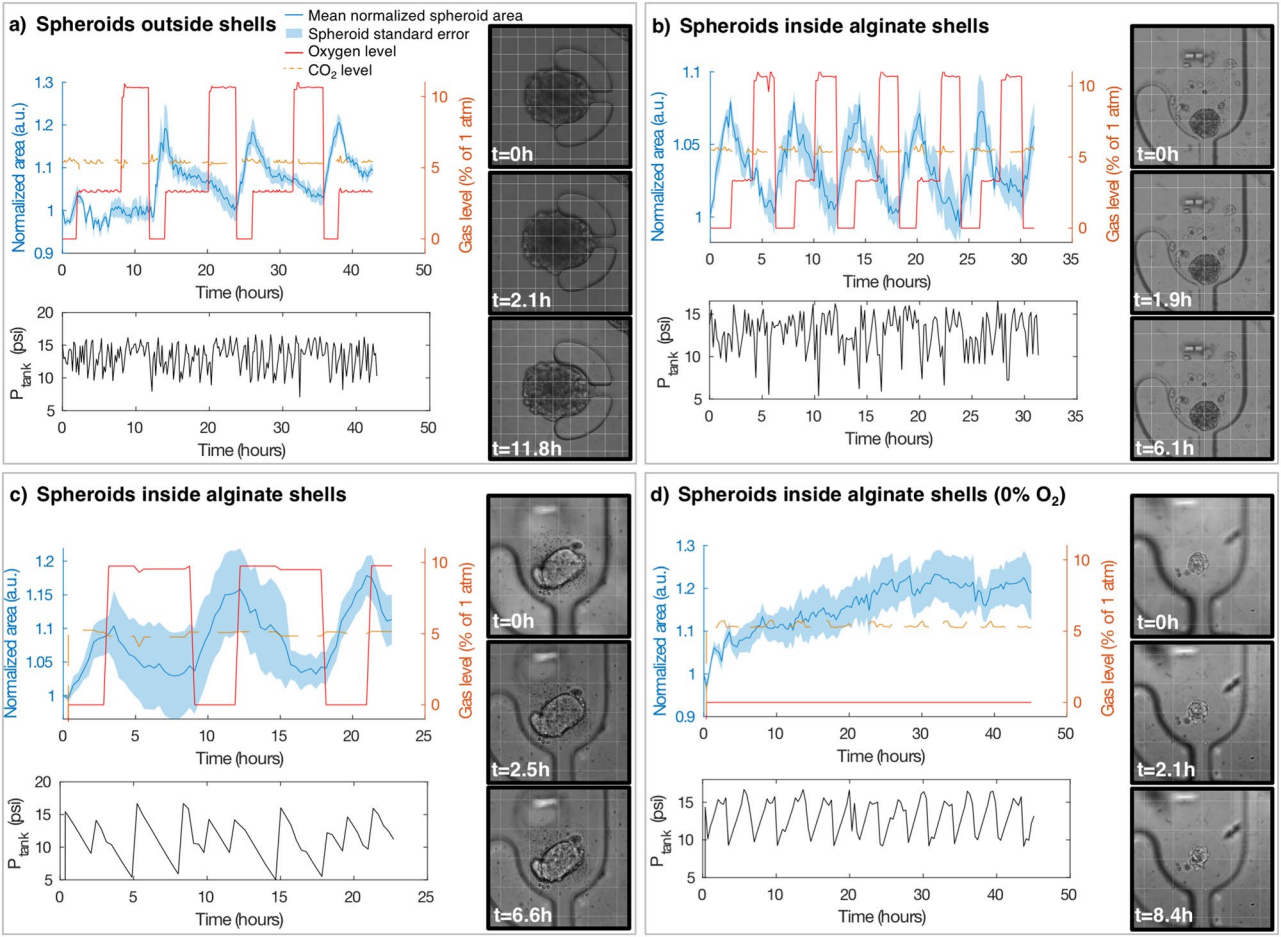
Cycling hypoxia-induced swelling of tumour spheroids (**a**) without and (**b,c**) within alginate shells in varying cyclic profiles, as well as (**d**) spheroids within shells exposed to chronic severe hypoxia (0% gaseous O_2_). (**a–d**) each depict the mean and standard error of the spheroid area over time for N = 5 (**a**,**c**), N = 6 (**b**), and N = 7 (**d**) spheroids. The measured pressure fluctuations of the gas supply tanks caused by emptying and refilling by the automated gas control system (P_tank_) are plotted below each spheroid area plot, showing that fluctuation of pressure inside the tanks does not correlate with swelling behaviour, as expected since the outputs of all tanks were regulated to 5 psi. Images of the spheroids at 3 time points during the experiment are shown, with different view magnifications to illustrate the spheroids of different sizes. A 100 μm grid is superimposed on each image for scale and to help visualize spheroid size changes. Cycling profiles in (**a–c**) were: 2 h 0% O_2_, 6 h 3% O_2_, 4 h 10% O_2_ (**a**), 2 h 0% O_2_, 2 h 3% O_2_, 2 h 10% O_2_ (**b**), 3 h 0% O_2_, 6 h 10% O_2_ (**c**).

We tracked spheroid size during cycling oxygen exposure via brightfield imaging and image segmentation, and observed a reversible spheroid swelling phenomenon in response to the changing oxygen levels (time-lapse videos depicting this swelling are shown in Supplementary Movie S1). We note that spheroids increase in size when exposed to 0% oxygen, and decrease in size when exposed to 3% and 10%. Figure 2 presents a summary of spheroid swelling behaviour in larger, mature (trapped 30 days after bead formation) spheroids outside of alginate shells (a) as well as smaller spheroids still within alginate shells, trapped 14 days (b) and 21 days (c) after bead formation). Each plot depicts the mean and standard error of size changes of N = 5–6 spheroids, as well as the oxygen levels supplied to the devices. (d) depicts the mean and standard error of the size changes of N = 7 spheroids grown at constant 0% oxygen, where it appears that the spheroid swelling behaviour slows down after longer 0% oxygen exposure periods. The size changes of three spheroids grown at 20% oxygen are presented in Fig. S1 of the Electronic Supplementary Information.

Across 3 separate experiments, 25 of 28 monitored spheroids visibly showed swelling and shrinkage in response to cycling hypoxia in time-lapse brightfield monitoring videos. It is important to note that spheroid size changes did not correspond with temporal changes in tank pressure (as expected since the outputs of all tanks were regulated to 5 psi) or CO_2_, as depicted in Fig. 2. We also note that alginate beads encasing small spheroids trapped within the same devices did not swell and shrink in response to the changing gas profiles (as depicted in Fig. S2 and Movie S2 of the Electronic Supplementary Information). As such, it appears that changes in oxygen level are the driving factor for the observed cyclic spheroid swelling behaviour. Time-lapse videos showing the dynamic swelling of representative spheroids are provided in the Electronic Supplementary Information (Movie S1), while time-lapse videos showing the swelling small spheroids trapped within non-swelling hydrogel beads are presented in Movie S2.

We observe from Fig. 2 that the larger spheroids no longer within alginate shells (having migrated out of the shells during their culture prior to trapping on-chip) appear to swell and shrink to a greater degree than the spheroids remaining within shells. The presence of the alginate shell around the spheroids may affect their swelling by inducing a confinement effect on the cells; confinement has been previously found to impact cellular growth, motility, and invasion in a microcapsule tumour spheroid model^48^. We note that many of the large spheroids that had migrated out of their shells were >270 μm (the approximate height of the cell culture channel) in diameter. It is likely that these spheroids were touching, and in some cases likely compacted, between the glass bottom and PDMS top of the cell culture channel. It is thus possible that the swelling measurement by area quantification may over-estimate size changes in these spheroids compared to smaller spheroids because the spheroid volume is constrained in Z. Future studies using optical sectioning microscopy to quantify spheroid volume could mitigate this shortcoming of measuring volume change via area. Analyzing the swelling for the first three swell-shrink cycles shows that the average per-cycle swelling for the spheroids outside of alginate shells (Fig. 2(a)) is 14 ± 7%, while those within shells is 7.1 ± 1.2% (Fig. 2(b)) and 11 ± 2% (Fig. 2(c)) (errors represent one standard deviation of the area change from the first three swell-shrink cycles of the average of 5–6 spheroids).

The results of Fig. 2 show that MCF-7 spheroids tend to swell and shrink in size in response to changing oxygen levels, with 0% oxygen inducing swelling behaviour within one hour after the change in oxygen level. Different spheroids appear to exhibit swelling behaviour to different degrees, with spheroid size and roundness (uniformity) having an effect on how readily the swelling can be observed in time-lapse videos.

Although this cyclic swelling behaviour, to our knowledge, has not been previously reported in the literature, there are known consequences of hypoxia that could drive its occurrence. The increase in size in response to low oxygen is consistent with cytoplasmic swelling within the packed cells in the spheroid, which can occur as a result of pH changes due to changes in cell metabolism as well as the failure of membrane ion pumps (due to inadequate adenosine triphosphate) and subsequent loss of membrane function in hypoxia/anoxia^49^. Cytoplasmic swelling could also be mediated by changes in the expression of aquaporins, which are water transport channel proteins in the cell membrane^50^. There is some evidence that tumour cell expression of aquaporin-1 may be correlated with hypoxia^51,52^. Hypoxic cell swelling has been linked with swelling-activated chloride currents, which are a mechanism of regulatory volume decrease and allow the cell to maintain function during changes in osmolarity^53^. Cellular swelling induced by hypoxia is potentially of interest because rapid cell volume changes can disrupt concentrations of species such as ions and enzymes within the cell responsible for homeostasis; as such, cell volume regulation via swelling-activated membrane ion channels is essential for cell function^54,55^. As cell volume changes can produce downstream effects in a wide range of functions such as proliferation, adhesion, and migration^55^, the effects of cycling hypoxia on spheroid volume could be important modulators of tumour cell behaviour.

Hypoxia can also stimulate migration of tumour cells^56^, and the observed increase in spheroid size could instead be due to reduction of cell-cell adhesion within the spheroid as the cells start to break apart from each other in the early stages of migration. Skiles *et al*. have reported regions of cytoplasmic swelling as well as loss of cellular cohesion (contacts) in fixed, sectioned spheroids grown at 1% and 2% oxygen for 4 days, which could also be consistent with our observed changes in spheroid size^57^. The loss of cellular cohesion observed by Skiles *et al*. could also be evidence of reduction of cell-cell adhesion, so we hypothesized that the changes in spheroid size that we observe as reversible swelling behaviour could be due to cytoplasmic swelling, reduction of cell-cell adhesion, or both. To test these two hypotheses for the mechanism of spheroid swelling behaviour (cytoplasmic swelling vs. loss of cell-cell contacts) we used optical sectioning microscopy to look inside the spheroid volume.

### Cells within the spheroid do not appear to disaggregate during swelling, suggesting that spheroid swelling is driven by swelling of individual cells

To ascertain whether the spheroid swelling observed in brightfield monitoring is driven primarily by swelling of individual cells or by disaggregation of the spheroid, we performed monitoring of the individual cells within the spheroids using two-photon microscopy and membrane staining. Three spheroids in each of two microfluidic devices were tracked using two-photon microscopy during exposure to 0% and 20% oxygen (chosen to reproduce severe hypoxia and *in vitro* normoxia (standard cell culture conditions), respectively). One microfluidic device was first maintained at 0% O_2_ and switched to 20% O_2_ after 224 minutes, while the other, serving as a control, was first maintained at 20% O_2_ and switched to 0% O_2_.

**Figure 3:**
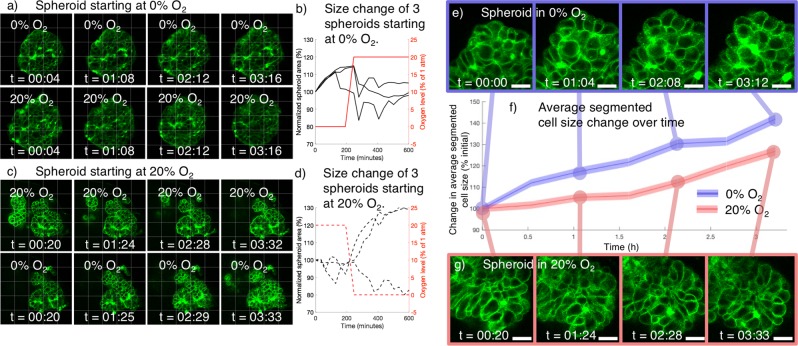
Two-photon monitoring of tumour spheroid membrane stain during on-chip exposure to 0% and 20% O_2_ shows spheroid swelling without disaggregation, as well as greater individual cell size increase under 0% O_2_. (**a**) Two-photon Z-slices of a representative spheroid starting at 0% O_2_ (top row) and switched to 20% O_2_ (bottom row) after 224 minutes. (**b**) Quantified spheroid swelling behaviour (via segmented transmitted light spheroid area) of spheroids starting at 0% O_2_ and switched to 20% O_2_ during monitoring. 3 of 3 spheroids increased in size during exposure to 0% O_2_ and ceased swelling after switching to 20% O_2_. (**c**) two-photon Z-slices of a representative spheroid starting at 20% O_2_ (top row) and switched to 0% O_2_ (bottom row) after 224 minutes. (**d**) Quantified spheroid swelling behaviour (via segmented transmitted light spheroid area) of spheroids starting at 20% O_2_ and switched to 0% O_2_ during monitoring. None of the spheroids swelled during exposure to 20% O_2_, and 2 of 3 spheroids swelled after switching to 0% O_2_. All times overlaid upon images indicate time in the current gas condition. 50 × 50 μm grid is superimposed in (**a–c**) to aid visualization of size changes. (**e**) Portion of a spheroid exposed to 0% O_2_ at varying times after initiating on-chip gas control. Gaps do not appear to form between the cells, despite spheroid swelling. (**f**) Plot of average cell size change over time, from segmented individual cells within spheroids exposed to 0% and 20% O_2_ during two-photon monitoring. Shaded coloured region denotes the standard error of the mean of all of the segmented cells in N = 3 spheroids under each condition. 1300–3200 total cells were measured at each time point for each condition to analyse the average cell size change. A Mann-Whitney U-test indicated a significant difference between the cell size distributions for the spheroids at 0% and 20% oxygen (p < 1 × 10^–4^) for all 6 timepoints t > 0. (**g**) Portion of a spheroid exposed to 0% oxygen at varying time points after initiating on-chip gas control. Scale bars on (**e–g**) depict 20 μm.

We used our observations of the brightfield swelling/shrinkage dynamics (Fig. 2) to inform our choices of experimental parameters in the 2-P study, selecting a 3 μm Z-slice spacing to offer sufficient temporal resolution to study the swelling/shrinkage dynamics of 6 spheroids (3 under each O_2_ profile). This large slice spacing presented limitations in that it confounded full 3D segmentation of the individual cells from the slice data; however, for this initial study we valued higher temporal resolution over higher spatial resolution. Representative Z-slice images and quantification of overall spheroid swelling are presented in Fig. 3(a–d).

Overall spheroid swelling at 0% O_2_ in 5 of the 6 monitored spheroids is evident both in the two-photon Z-slices and the quantification of segmented spheroid area in Fig. 3(a–d). 3 of the 3 monitored spheroids in the first microfluidic device swelled immediately upon starting the 0% O_2_ gas and monitoring, while 2 of the 3 monitored spheroids in the second microfluidic device swelled after switching to 0% O_2_ after 224 minutes. The individual Z-slices show the membrane outlines of the individual cells that make up the spheroid, and it does not appear that gaps between cells form during the experiment in the monitored spheroids. This suggests that swelling of individual cells, rather than spheroid disaggregation, is the driving phenomenon behind the observed spheroid swelling.

### Individual cells within the spheroid appear to swell under exposure to 0% oxygen, driving spheroid swelling behavior

To test the hypothesis that swelling of individual cells was the driving factor behind the observed spheroid swelling behaviour, we segmented individual cells from the two-photon images of 3 spheroids in each of the 20% and 0% oxygen conditions to measure the change in average cell area. The segmentation protocol is described in the Supplementary Information, and cropped images of example spheroids, alongside this average cell size data, are presented in Fig. 3(e–g).

It appears from Fig. 3(e–g) that the cells in the spheroid exposed to 0% oxygen increase in size earlier, and at a faster rate, than those in the spheroids exposed to 20% oxygen, suggesting that the cell swelling is oxygen-dependent and likely affirming that swelling of individual cells drives the spheroid swelling behaviour. Although the overall spheroids exposed to 20% oxygen did not show appreciable swelling (as depicted in Fig. 3(d)), we do measure an increase in the average individual cell size in the spheroids under this condition. This observation could be due to artefacts from the segmentation process (such as reduced signal to noise ratio or stain internalization at the later time points), or due to actual cell swelling caused by the membrane staining or monitoring process. Regardless, the significant difference in swelling observed between the 20% oxygen control and 0% oxygen spheroids (Mann-Whitney U-test p < 1 × 10^–4^ for all 6 timepoints t > 0) does suggest that 0% oxygen induces cell swelling within the spheroids, and this cell swelling likely drives the observed spheroid swelling behaviour. Future work could examine intra-spheroidal spatial heterogeneity in the swelling behaviour at the single-cell level. As large spheroids (400–600 μm diameter) tend to form a necrotic core^6,58^ (which we would not expect to reversibly swell as the cells are no longer viable), and there exists an oxygen gradient within spheroids, spatial variation in the reversible swelling behaviour might be expected.

### The microfluidic platform facilitates monitoring of doxorubicin penetration under tunable hypoxic profiles, and demonstrates that cycling hypoxia may affect doxorubicin accumulation in tumour spheroids

To investigate whether different oxygen conditions may affect the accumulation of anticancer treatments, we again leveraged the combined dynamic monitoring and oxygen control capabilities of our microfluidic system by using two-photon optical sectioning microscopy to measure accumulation of doxorubicin, an intrinsically fluorescent cytotoxic anticancer treatment. Doxorubicin is an anthracycline antibiotic anticancer drug that intercalates DNA and binds to DNA-associated enzymes to cause cytotoxic damage to cells. It preferentially affects dividing cells but can also affect interphase cells^59^. Doxorubicin has been used in the treatment of many types of cancer, including breast cancer, since its development in the 1960s, and it has been identified as one of the most effective anticancer drugs^60^. Doxorubicin is intrinsically fluorescent; however, its fluorescence serves as a conservative estimate of uptake because its fluorescence is quenched when it intercalates DNA^61^. Optical sectioning microscopy modalities such as confocal and two-photon are commonly used to measure drug uptake^62^, both of small molecule^63,64^ and liposomal/nanoparticle^65–68^ formulations of drugs. In this study, we are, for the first time, monitoring spheroid drug uptake with two-photon microscopy throughout the course of a 72 h treatment under controlled time-varying hypoxic conditions.

Separate microfluidic devices with loaded tumour spheroid cultures within core-shell hydrogel beads were maintained at 0% O_2_ (chronic severe hypoxia), 20% O_2_ (*in vitro* normoxia), and a cycling oxygen profile of 4 h at 0% followed by 4 h at 20%. All gas conditions contained 5% CO_2_ and the devices were maintained in the same microscope stage-top incubator system. The tumour spheroids were perfused with 10 μM doxorubicin for 72 h while monitoring with two-photon microscopy, acquiring a Z-stack (with 4 μm Z-slice spacing) of each spheroid every ~30 minutes (the microscope was left running continuously, imaging the 15 monitored spheroids across the 3 devices sequentially before going back to the first). Spheroid size changes were quantified by segmenting representative transmitted light slices acquired at a focal depth 40 μm into the spheroid to quantify projected spheroid area. The average doxorubicin fluorescence intensity in the segmented region of this representative slice was quantified as a measure of doxorubicin accumulation. Average fluorescence in the spheroid region (rather than total summed intensity) was chosen as the relevant metric to mitigate the impact of spheroid size heterogeneity. The segmentation and average fluorescence intensity calculation was repeated for every slice in the Z-stack to yield a representation of doxorubicin accumulation throughout the whole spheroid.

**Figure 4:**
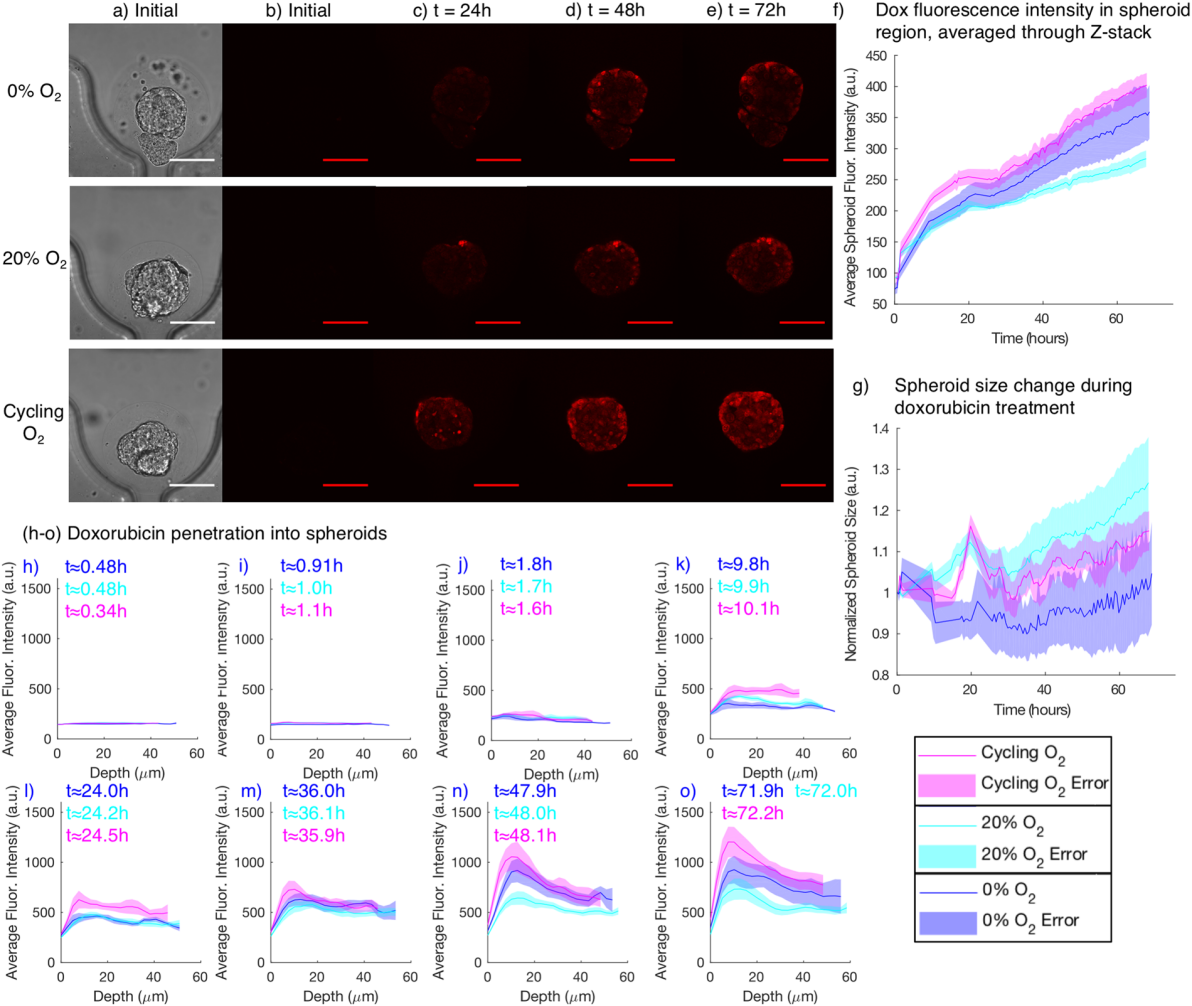
Monitoring doxorubicin (dox) accumulation and spheroid size change during treatment under *in vitro* normoxia (20% O_2_), chronic severe hypoxia (0% O_2_), and cycling hypoxia (cycling gas profile with 4 h 0%, 4 h 20% O_2_). (**a**) Example transmitted light images used for spheroid segmentation. (**b**) Doxorubicin fluorescence at the beginning of the experiment, prior to starting the doxorubicin flow. Almost no fluorescence is visible. (**c–e**) Two-photon doxorubicin fluorescence images after 24, 48, and 72 h of doxorubicin treatment. (**f**) Quantified average doxorubicin fluorescence intensity in the spheroid region (the total summed pixel intensity in the segmented region divided by its pixel area). (**g**) Quantified segmented spheroid area change during the 72 h treatment, for spheroids in each of the tested oxygen conditions. Doxorubicin accumulation is higher under cycling hypoxia than under chronic severe hypoxia and normoxia. (**h–o**) Doxorubicin penetration, visualized as average doxorubicin fluorescence intensity vs. depth into the spheroid, for different oxygen conditions, at varying times in the experiment. Imaging the complete set of spheroids in all three conditions took ~30 minutes, so the average time for each set of images of each condition is reported. Issues with the microscope stage automation reduced temporal resolution over the first ~24 h of the experiment. Shaded regions depict standard error of the mean for N = 5 (cycling), 6 (20%), 4 (0%) spheroids.

Figure 4 presents images of representative spheroids at the three investigated oxygen levels at different times during the 72 h doxorubicin exposure, and also presents quantification of spheroid size and doxorubicin accumulation during the experiment for the three oxygen conditions. Supplementary movies S3–S5 depict two-photon monitoring of the doxorubicin fluorescence intensity in single Z-slices for a representative spheroid from each condition.

Cyclic spheroid swelling behaviour is evident in the spheroids exposed to cycling oxygen in Fig. 4(f), and not in the spheroids exposed to chronic severe hypoxia (0%) or *in vitro* normoxia (20%). The doxorubicin accumulation of the spheroids exposed to the cycling oxygen profile is notably higher and faster than that of the spheroids exposed to *in vitro* normoxia (20%). This increased accumulation is also visible in the slice images shown in Fig. 4(a–e): both higher overall intensity and increased penetration into the spheroid (higher fluorescence intensity further from the edges of the spheroid) are visible after 24 h doxorubicin treatment in the spheroid exposed to cycling oxygen than the other two representative spheroids. This higher accumulation in spheroids exposed to cycling hypoxia suggests that reoxygenation cycles in hypoxic culture (such as those that may be unintentionally introduced by media exchanges) could affect the results of *in vitro* drug uptake studies.

The expression of the key multi-drug resistance transport protein P-glycoprotein has been previously found to be upregulated under hypoxia, and downregulated by reactive oxygen species such as those that can be generated by cycling hypoxic profiles^69^. As P-glycoprotein can act as an efflux pump, affecting doxorubicin uptake by the cell, modulation of P-glycoprotein expression by reactive oxygen species generated by cycling hypoxia could be one potential hypothesis for the mechanism driving the increased doxorubicin accumulation that we observe. Interestingly, the P-glycoprotein has also been suggested to play a role in cell volume regulation by potentially regulating swelling-activated anion channels^70,71^, which have also been found to be induced by hypoxia via hypoxic swelling^53^. Other potential hypotheses for the increase in doxorubicin accumulation that we observe under cycling hypoxia include (1) oxidative stress induced by the hypoxia-reoxygenation cycles may impact the tumour cells’ drug efflux properties, (2) cell volume regulation or the mechanical cues of the swelling-shrinkage process may impact drug efflux, or (3) the swelling-shrinkage behaviour may alter the mass transport properties of the tumour spheroids. Future work using this platform will leverage its ability to monitor spheroid behaviour during the dynamic hypoxia treatment, as well as during subsequent fixation, immunofluorescence staining, and endpoint imaging to correlate data from the same spheroids and identify the mechanism for this observed differential uptake.

We unexpectedly observe higher doxorubicin accumulation in spheroids under chronic severe hypoxia than in spheroids under *in vitro* normoxia. This is inconsistent with previous studies, which tend to show lower doxorubicin efficacy and accumulation at low oxygen levels, although there are also discrepancies between these previous studies. Using standard 96-well plates and hypoxic incubators, Strese *et al*.^72^ found that the IC_50_ of monolayer MCF-7 cells in anoxia/severe hypoxia (0.1% oxygen) was greater than 2.1 times that of cells in normoxia (i.e., the drug became less effective in anoxic conditions). They also found a dependence of the IC_50_ on the level of hypoxia: the doxorubicin IC_50_ of MCF-7 cells in moderate hypoxia (1% oxygen) was 0.59 times that of cells in normoxic (20–21% oxygen) conditions (i.e., the drug became more effective in hypoxia)^72^. In another study, Doublier *et al*.^73^ found that hypoxic exposure (3% oxygen) led to significantly less drug accumulation in MCF-7 cells after an 18-hour treatment with 3 μM doxorubicin (indicative of doxorubicin resistance at 3% oxygen, in contrast to the doxorubicin sensitivity at 1% oxygen reported by Strese *et al*. on the same cell line). The authors also showed less drug accumulation in tumour spheroids than monolayer cells, and attributed this doxorubicin resistance in part to the activation of HIF-1α, which can occur at 3% oxygen. They did not compare accumulation in spheroids under normoxic and hypoxic conditions, but it is worth noting that our results show initially higher accumulation in spheroids under 20% O_2_ than those under 0% O_2_, with 0% O_2_ accumulation overtaking that of the normoxic spheroids after ~24 h (after the 18 h doxorubicin exposure period of Doublier *et al*.). It is thus possible that our results may actually be consistent with this previous work with shorter drug treatment periods, and the spheroids’ drug uptake behaviour under varying oxygen conditions may be dynamic over longer-term exposures. The mechanism for this dynamic behaviour and our higher long-term measured accumulation at 0% than 20% O_2_ is unclear, and may benefit from future studies.

The representative slice images of Fig. 4(b–e) also show the heterogeneity in doxorubicin uptake within the cells that make up each spheroid. As quantified in Fig. 4(h–o), we observe an intensity gradient from the spheroid edge to centre (with higher signal at the spheroid edge); this gradient is likely due to the combination of a diffusive gradient in drug uptake, microenvironmental gradients such as pH, as well as optical scattering of the spheroid (as assuming a roughly spherical spheroid, the optical path length is longer near the centre of the spheroid than at its edges). Rather than showing only a steady gradient in fluorescence intensity from the edge to centre of the spheroid, we also observe unpredictable heterogeneity in uptake: images under each oxygen condition show some cells with higher doxorubicin fluorescence intensity immediately beside cells with much lower intensity. Heterogeneity in doxorubicin fluorescence has been previously measured in spheroid flow cytometry data and attributed primarily to diffusional limitations, although heterogeneity in uptake was observed even in cells from disaggregated spheroids^74^. P-glycoprotein-mediated multidrug resistance has been found to reduce doxorubicin uptake to 19% of wild-type levels, suggesting that heterogeneity in P-glycoprotein expression could be one hypothesis for the mechanism of the observed uptake heterogeneity^75^. Another potential hypothesis for the observation of brightly fluorescent cells beside dimmer cells could be cell migration within the spheroids. We observe cell migration in our two-photon time lapse videos (Supplementary movies S3–S5) during the doxorubicin treatment, so it is possible that cells at the spheroid surface could migrate inwards after accumulating considerable amounts of doxorubicin. Doxorubicin accumulation in cells has also been observed to be dependent on extracellular and intracellular pH, with highest accumulation at high extracellular and intracellular pH^76^; this pH dependence could form another hypothesis for the observed heterogeneity in uptake. pH-dependent differences in measured doxorubicin signal are likely to be due to differences in accumulation, as doxorubicin fluorescence is thought to be only weakly pH dependent^76^. The observed intra-spheroidal doxorubicin uptake heterogeneity demonstrates why optical sectioning microscopy monitoring with single-cell resolution in 3D cultures can be beneficial in understanding tumour model response to stimuli such as anticancer treatment.

From the size monitoring data of Fig. 4(g) we observe higher spheroid size increase during doxorubicin treatment under 20% O_2_ than under 0% O_2_. We hypothesize that this observation may be due to the combination of multiple factors impacting the size of the spheroids: (1) hypoxic swelling, (2) proliferation, and (3) swelling induced by the toxicity of the doxorubicin treatment. Doxorubicin has been previously observed to induce oncosis (cell death characterized by swelling^77^) in subpopulations of neuroblastoma cells and cardiomyocytes^78^, as well as in endothelial cells^79^ and follicular cells during chemotherapy^80^. We also observe that doxorubicin appears to change reversible spheroid swelling behaviour, with spheroids treated with doxorubicin exhibiting larger and more variable volume changes than control spheroids under a cycling oxygen profile (as depicted in Fig. S4 of the Electronic Supplementary Information). As P-glycoprotein-mediated doxorubicin efflux has been found to be inhibited by cell swelling^81^, future study into the mechanisms of hypoxic swelling and potential links to drug uptake could lead to new insights into the role of cycling hypoxia in treatment efficacy.

Using optical sectioning microscopy allows us to quantify doxorubicin penetration into the spheroid, as well the overall uptake presented above. Although there may be short term dynamics that were not observed in this experiment, the long-term monitoring that we present is relevant to longer-term drug efficacy studies and future work could assess shorter-term dynamics of the drug response. We obtain a full Z-stack of each spheroid at each time during the monitoring experiment, so we are able to quantify average fluorescence intensity at varying distances into the spheroid. An example of this kind of measurement is presented in Fig. 4(h–o), where we plot average doxorubicin fluorescence intensity vs. depth into the spheroid (distance from the segmented edge of the spheroid in a Z-slice 40 μm into the spheroid). The initial rise in fluorescence intensity from ~0–10 μm is due to over-estimation of the spheroid edge by the transmitted light segmentation algorithm; future studies could include a reference fluorophore to label the cells for more accurate segmentation of each Z-slice. We again observe higher doxorubicin uptake deep into the spheroids in the spheroids exposed to the cycling oxygen profile, consistent with the overall spheroid uptake results described above. We also initially see higher uptake and deeper penetration in the spheroids exposed to 20% O_2_ than those exposed to 0% O_2_, and subsequently see higher uptake at all depths in the spheroids exposed to 0% O_2_ than those exposed to 20% O_2_, consistent with the overall spheroid uptake results which show the 0% spheroids overtaking the 20% spheroids after ~24–36 h. Future work will study whether other classes of cancer treatment, or other cancer treatment formulations (such as nanoparticle formulations of doxorubicin^82^) have similar dependence on oxygen levels surrounding tumour spheroids.

## Discussion

We have demonstrated a microfluidic platform capable of time-lapse monitoring of an array of tumour models exposed to dynamic, time-varying, and precisely controlled oxygen conditions during anticancer treatment. Combining dynamic optical sectioning microscopy monitoring with precise temporal oxygen control allows us to demonstrate, for the first time, that tumour spheroids dynamically swell and shrink under a time-varying oxygen profile, and that this swelling behaviour appears to be driven by the swelling of individual cells within the spheroid. Using optical sectioning microscopy allows us to optically access cells positioned at a range of positions within the heterogeneous tumour spheroid environment, allowing us to measure for the first time the dynamics of tumour model response to anticancer treatment under chronic and cycling hypoxia as well as normoxia.

The oxygen and nutrient consumption of tumour spheroids drive formation of diffusion gradients in oxygen and nutrient levels from the spheroid surface to its core^83,84^. As such, by precisely controlling oxygen at the spheroid surface, our microfluidic platform modulates the spatiotemporal dynamics of the oxygen gradients that naturally form within spheroids. By supplying temporally varying oxygen profiles to the spheroid surface we can mimic either (1) oxygen levels within an avascular tumour region adjacent to an intermittently perfused blood vessel, or (2) the effects of media or drug changes (accidental reoxygenation cycles) in traditional spheroid culture in hypoxic incubators. Both of these modeled systems may be relevant in drug development or the mechanistic study of tumour behaviour.

We anticipate that the microfluidic platform we have described, combined with fixation, on-chip tissue clearing^85^, and antibody probing at the study endpoint, will facilitate the testing for the mechanisms driving dynamic tumour model behaviour in response to microenvironmental changes or drug treatment. Future coupling with higher-throughput optical sectioning microscopy systems such as kHz framerate two-photon tomography^86^ or light sheet microscopy^87–89^ could permit precise volumetric changes of high numbers of spheroids to be quantified during monitoring without compromising temporal resolution. The platform could also be used to assess new strategies for overcoming hypoxic resistance to treatment. The platform’s ability to monitor the same tumour spheroids during hypoxic exposure, anticancer treatment, fixation, tissue clearing, and future molecular probing opens the door for a wide range of high-content imaging studies not possible using traditional technologies. We believe there would be significant benefit in future studies to identify the molecular mechanism and implications of the dynamic spheroid swelling that we report here, as well as to identify the temporal response profile of tumour spheroids to varying periods of hypoxia that modulate sensitivity to anticancer treatment. The understanding gained from these studies could aid in better understanding of disease progression in cancer as well as in the design and selection of more effective cancer treatments.

## Methods

### 3D cell culture

MCF-7 breast tumour spheroids within hydrogel beads were generated in a flow-focusing process as previously described^47^. Briefly, MCF-7 cells were cultured in DMEM/F-12 (Sigma-Aldrich, St. Louis, MO, USA) supplemented with 5% FBS (Sigma-Aldrich, St. Louis, MO, USA) and 1X Antibiotic-Antimycotic (Invitrogen, Carlsbad, CA, USA). After rinsing in PBS and detaching in 0.05% trypsin-EDTA (Invitrogen, Carlsbad, CA, USA), the cells were resuspended at a concentration of approximately 1 × 10^7^ cells/mL in a chilled solution of 180 μL High Concentration Rat Tail Collagen I (Cat# 354249, Corning, Corning, NY, USA, 8–11 mg/mL stock concentration), 180 μL Matrigel^®^ Basement Membrane Matrix (Cat#354234, Corning, Corning, NY, USA, 8–12 mg/mL stock concentration) and 140 μL 2% Manucol LKX alginate (FMC Biopolymer, Norway) in Ca/Mg(−) HBSS (Sigma-Aldrich, St. Louis, MO, USA), using cooled pipette tips and tubes. The solution was neutralized with the addition of 7 μL NaHCO_3_ (Invitrogen, Carlsbad, CA, USA), and kept on ice throughout the process to prevent collagen and Matrigel^®^ gelation. The cell solution was supplied to the central inlet of a microfluidic flow-focusing device, and sheath flows of 40 mM CaCO_3_ in 2% alginate solution were supplied to either side. Droplets of the flow-focused aqueous phase were formed in a microfluidic droplet-generating chip using a mineral oil continuous phase acidified with 0.1% acetic acid. 1% (w/v) Span80 was added to the continuous phase to prevent droplet coalescence prior to gelation.

After collection, beads were washed twice in incomplete media and subsequently cultured in the same complete DMEM/F-12 as the monolayer cells. The cell-laden beads (seeded with ~5 disperse cells per bead at the start of culture) were cultured in non-adherent T75 culture flasks at 37 °C with 5% CO_2_ in a standard cell culture incubator until the cells had proliferated into tumour spheroids within the beads (2–3 weeks). Once tumour spheroids had formed, the beads were loaded into the microfluidic trapping devices for on-chip studies.

### Microfluidic device fabrication

The 3-layer oxygen control microfluidic devices consist of (1) a bottom layer with a cell culture channel containing an array of C-shaped hydrodynamic bead traps (each with a flap to aid in bead retention), surrounded by hydration and gas control channels, (2) a hydration layer, through which PBS is perfused to mitigate media evaporation into the flowing gas used for oxygen control, and (3) a gas control layer. The devices were fabricated using multilayer soft lithography as described in our previous work^45^. Device fabrication and preparation details are provided in the Electronic Supplementary Information.

### Bead loading

To load the spheroid-laden beads into the microfluidic devices, the cell culture channels were first flushed with 1.5 mL of complete DMEM/F-12 to prevent alginate dissolution in the Ca^2+^-free PBS. Beads, suspended in complete DMEM/F-12, were loaded into a 3 mL Luer-lock syringe, connected to the cell culture channel inlet, and then allowed to gravitationally settle in the syringe tip with the tubing blocked with a tubing clamp for approximately 20 s. This step yields a high concentration of beads to improve trapping efficiency. After settling, the tubing was unblocked and beads were perfused into the cell culture channel for trapping. After trapping was completed, the tubing was again blocked, syringes switched, and complete media was perfused through the channel to rinse away untrapped beads. Ratchet tubing clamps were used to block the flow when changing fluids or moving the chips to reduce backflow, which could dislodge the beads from the hydrodynamic traps.

### Tumour spheroid culture and monitoring

NBeads were cultured on chip in a microscope stage-top incubator (Custom Chamlide TC CU-109, Quorum Technologies, Guelph, Canada), at 37 °C while monitoring with brightfield and/or fluorescence microscopy (Nikon TE2000U) or two-photon fluorescence microscopy (Olympus FV1000 with MaiTai HP DeepSee multi-photon source). Two-photon studies employed a 25X water dipping objective with correction collar for coverslip thickness (Olympus XLPLN25XWMP, NA = 1.05, working distance 2 mm). For two-photon studies, the incubator base was used, while the microfluidic chips were inverted and the large cover glass was used to seal the chamber such that the microfluidic devices could be imaged using the upright microscope through the cover glass. The temperature within the chamber was verified with a PT 100 temperature probe (LCI TS-B, Quorum Technologies, Guelph, Canada) affixed to the glass substrate of the microfluidic systems.

The cell culture channels of the microfluidic devices were perfused with completed cell culture media (DMEM/F-12 supplemented with 5% FBS and 1X anti-anti) at a flow rate of 0.5 μL/min. Hydration channels were perfused with PBS at a rate of 0.5 μL/min. All media and PBS were degassed before perfusion to reduce bubble formation in the warm tubing and channels. Media and PBS were heated to 50 °C for 15–20 minutes in a water bath and then the solutions were degassed in a desiccator for 20 minutes while the fluid was still warm. The solutions were then very carefully taken into syringes without forming bubbles, and connected to the inlet tubing for perfusion.

### Two-photon monitoring of single-cell size changes within spheroids

For single-cell monitoring within tumour spheroids, the spheroids were stained with CellMask^TM^ Green membrane stain (Thermo Fisher C37608) prior to loading into the microfluidic devices and imaging. Spheroids were first rinsed 3X in Hank’s Buffered Saline Solution (HBSS, Invitrogen) without Ca^2+^ and Mg^3+^ via gravity settling, and then resuspended in HBSS containing the stain solution at a 500X dilution. The HBSS rinse step removed the alginate shells surrounding the spheroids prior to staining by depletion of Ca^2+^ ions from the Ca^2+^-gelled alginate. We used HBSS for staining and loading because stained spheroids in incomplete media tended to adhere to the sides of the Falcon tubes and pipette tips used for staining, resulting in high spheroid loss. The spheroids were incubated in the dark at 37 °C for 1 h to permit stain penetration before rinsing 3X in HBSS and loading into the microfluidic devices for culture following the loading and culture protocol described above. Spheroids were loaded into two microfluidic devices and simultaneously exposed to different oxygen conditions within the same culture and imaging setup. The scanning-laser two-photon microscope (Olympus FV1000 with MaiTai HP DeepSee multi-photon source and XLPN25XWMP 25X water dipping objective) was left running continuously, sequentially imaging spheroids from both microfluidic devices. The two-photon excitation wavelength for this study was 810 nm, yielding diffraction-limited x-y and z-resolutions of *ω*_*xy*_ = 0.18 μm and *ω*_*z*_ = 0.60 μm, respectively. Each spheroid was imaged every 20 minutes, acquiring Z-stacks approximately halfway into each spheroid at a 3 μm z-stack slice spacing.

### Anticancer treatment studies

For drug studies, doxorubicin (Sigma Aldrich D1515–10MG dissolved in DMSO and aliquoted prior to freezing to avoid freeze-thaw cycles) was added to the completed media immediately before perfusion (after degassing). All doxorubicin concentrations and vehicle controls were balanced to the same concentration of 1000X dilution from the stock solution (0.1% DMSO final). Doxorubicin is a cytotoxic anthracycline antibiotic anticancer drug, and appropriate personal protective equipment and disposal guidelines were followed. For the two-photon monitoring spheroid study, a drug concentration of 10 μM doxorubicin was used. Drug-containing media was supplied at a flow rate of 10 μL/min for 10 minutes immediately after the drug-containing media syringe was connected to the microfluidic system to bring the doxorubicin through the tubing before lowering the flow rate to 0.5 μL/min for the remainder of the study. The microscope was left running constantly during two-photon imaging of the intrinsic doxorubicin fluorescence, imaging each spheroid every 40 minutes at a 4 μm Z-stack slice spacing.

### Oxygen control

Constant oxygen concentrations were provided to the devices by perfusing the microfluidic gas control channels with gas from custom-mixed gas cylinders with known oxygen levels and 5% CO_2_ (Praxair, Delta, Canada). Time-varying oxygen profiles were provided using a system built from an Arduino microcontroller controlling an array of solenoid valves to switch between cylinders of mixed gases. We have used Arduino-based system to switch between pre-mixed gas cylinders as well as mix gas cylinders from air, nitrogen, and CO_2_ (with reference sensors for validation and feedback) as described in our previous work^45^. All gas mixtures contained 5% CO_2_.

### Image processing

Presented images were brightness and contrast-adjusted in Fiji^90^ for visibility by setting maximum and minimum levels to the bounds of the image histogram. OME’s Bio-Formats^91^ plugin was used to read in the confocal image stacks to Fiji.

Segmentation of brightfield, fluorescence, two-photon fluorescence, and transmitted light images to measure spheroid size was performed using custom scripts written in MATLAB^®^ (Mathworks). Details regarding the image analysis methods are provided in the Electronic Supplementary Information and Fig. S4.

## Supplementary information

Supplementary information document

Movie S1

Movie S2

Movie S3

Movie S4

Movie S5

## Data Availability

The datasets included in the current study are available from the corresponding authors upon reasonable request.
