# Supplementary material for: Long-term monitoring in a microfluidic system to study tumour spheroid response to chronic and cycling hypoxia
Source: Sci Rep. 2019 Nov 28;9:17782. doi: 10.1038/s41598-019-54001-8 (PMC6883080; doi:10.1038/s41598-019-54001-8)
Supplement: Supplementary file 1 — Supplementary information document [file 41598_2019_54001_MOESM1_ESM.pdf]

Electronic Supplementary Information for:

Long-term monitoring in a microfluidic system to study tumour spheroid response to chronic and cycling hypoxia

Samantha M. Grist*[1], S. Soroush Nasseri[1], Loïc Laplatine[1], Jonathan C. Schmok[1], Dickson Yao[1], Jessica Hua[1], Lukas Chrostowski[1], and Karen C. Cheung*[1]

[1] Department of Electrical and Computer Engineering, The University of British Columbia.

Supplementary Methods

Microfluidic device Fabrication

SU-8 3050 photoresist (Microchem, Westborough, USA) on silicon wafer (University Wafer, South Boston, USA) masters were fabricated with resist thicknesses of 270 µm (cell culture layer 1), 80 µm (hydration layer 2), and 270 µm (gas control layer 3). For improved mould longevity, a polyurethane (Smooth-on, Macungie, USA) mould master was fabricated for layer 3 using an intermediate poly(dimethylsiloxane) (Sylgard® 184 PDMS, Paisley Products of Canada, Scarborough, Canada) mould (2-part polyurethane was cast from the first PDMS moulded to the SU-8-silicon mould).

Sylgard® 184 PDMS was cast to the layer 3 (top gas control layer) mould to a thickness of approximately 8 mm. The same PDMS was spin-coated on the silicon wafer mould for layer 2 using a 3-step spin program (5s at 50 RPM, 10s at 100 RPM, and 30s at 270 RPM) and on the silicon wafer mould for layer 3 (10s at 50 RPM, 10s at 100 RPM, and 30s at 200 RPM). These spin protocols typically yielded PDMS layer thicknesses between the channels of approximately 200-250 µm. All PDMS layers were cured at 65°C for at least 2-3 hours prior to bonding. Layer 3 was demolded and input and output holes punched (using a 0.5 mm Harris Uni-Core® biopsy punch, no longer available) before bonding it to the spin-coated surface of layer 2 using a 75s air plasma exposure at a pressure of 600-800 mm Hg. After bringing the two PDMS surfaces into contact and pressing gently to remove bubbles, the bonded device was placed into a 65°C oven for 1 hour before repeating the bonding process to bond layer 1 to the bonded layers 2-3. After all 3 layers were bonded, all inlet and outlet holes were punched, and the individual devices were diced, cleaned, and inspected for defects with microscopy before bonding.

PDMS microfluidic devices were air plasma-bonded to large (76x89 mm), 0.19-0.25 mm thick microscope cover glass slides (Ted Pella, Redding, CA, USA). Up to 6 microfluidic devices could be bonded to the same microscope cover glass for use in a single experiment using a 75s plasma exposure followed by overnight baking in an oven at 65°C to ensure high bond strength and complete PDMS curing prior to degassing and using the devices. Throughout setup and use, thick glass slides (102x76x1 mm, Ted Pella, Redding, CA, USA) were used as supports for the fragile cover glass substrates, except when using two-photon microscopy requiring thin cover glass.

Microfluidic device preparation

Devices were checked for leakage and degassed by first flowing 70% ethanol through all channels using manual syringe-driven flow through Tygon® microbore tubing (Cole-Parmer, Vernon Hills, USA) interfaced with 22G fluid dispensing tips (Nordson EFD, East Providence, USA). Luer-lock fluid dispensing tips were used to interface the syringe to the tubing, while fluid dispensing tips with the plastic Luer portion carefully removed were used to interface the tubing to each microfluidic input and output. After checking for leakage, all channels were filled with deionized water and the device was immersed in deionized water in a 1L beaker and degassed in a desiccator for at least 1 hour with

a thick glass slide underneath (for support) and on top (to prevent flotation) of the bonded chips in the beaker. Immersed devices were then briefly sonicated to release formed bubbles and kept in a 65°C oven overnight to complete PDMS hydration, with the beaker top covered completely in aluminum foil to mitigate evaporation.

After degassing, Tygon® microbore tubing was connected to all device inputs and outputs via 22G blunt needles (removed from Luer-lock fluid dispensing tips). The device remained underwater in a large petri dish until all fluidic I/Os were connected. All tubing for fluidic inputs was connected to a syringe filled with either 70% ethanol or PBS and filled with fluid prior to connection to avoid introducing bubbles into the degassed device. Approximately 500 µL of 70% ethanol was flowed through the cell culture channel to help to sterilize it before switching to sterile PBS. Syringes were carefully switched at the tubing end at all fluid exchanges to avoid introducing bubbles, and plastic ratchet tubing clamps (Cole-Palmer, Vernon Hills, IL, USA) were used to block the tubing during syringe exchange (to prevent bubble introduction or backflow). All channels were flushed with 1 mL of sterile PBS (for hydration channels) or 2.5 mL of sterile PBS (for the cell culture channel) before loading the spheroid-laden beads. The outlet tubing for the fluid channels was placed into two 50 mL Falcon® tubes (one for the cell culture channels and one for the hydration channels) with a hole in the lid and sealed with Parafilm®, to collect the perfused fluid.

Image processing

Spheroids were segmented from brightfield and transmitted light images by intensity thresholding or edge detection (depending on the spheroid light transmission properties) followed by morphological open and close operations, a fill operation to fill holes in the detected spheroid, and removal of segmented regions smaller than a predefined spheroid size threshold. In some cases, images for segmentation were filtered by a median filtering operation or a Gaussian blur filter prior to thresholding or edge detection to remove noise and interference patterns surrounding refractive index discontinuities in transmitted light images. The software allowed the user to manually draw a mask region to ensure that portions of the microfluidic trap were not segmented in the spheroid segmentation process. If automatic segmentation was not able to detect the spheroids due to lighting issues during the time-lapse or due to the presence of residual $CaCO_3$ particles within the alginate, manual segmentation of the spheroid area was used. Spheroids were segmented from fluorescence images by intensity thresholding followed by the same morphological open, close, fill, and small object removal operations.

To ascertain whether swelling of individual cells within the spheroids was driving spheroid swelling behaviour, we segmented two-photon images of spheroids with fluorescently-stained cell membranes. This segmentation was performed by a semi-automated segmentation script using the Cellsegm MATLAB toolbox[1] for membrane stain segmentation. Individual slices of two-photon fluorescence Z-stacks were segmented using the surface stain segmentation functionality of the Cellsegm toolbox, which uses smoothing and ridge enhancement prior to finding local image minima to use as seeds for a Watershed segmentation. We observed good segmentation results for early imaging time points; however, the long monitoring periods (>3h) yielded lowered signal-to-noise ratios as well as stain internalization at the later time points, which confounded automated segmentation. A MATLAB script incorporating the Cellsegm library was thus written to permit manual user refinement of the generated seeds as well as the final segmentation. The segmentation was performed on 10-12 Z-slices from each spheroid, taking the mean of the segmented cell areas in each spheroid at each time point. Between 1300 and 3200 cells were analyzed per experimental condition at each time point. All manual segmentation was performed by researchers blinded to the experimental conditions of each spheroid.

To quantify doxorubicin uptake, transmitted light images of the spheroid were segmented to ascertain the spheroid region, and then the average doxorubicin fluorescence intensity (total fluorescence in the segmented spheroid region divided by its area) was quantified as a metric of

doxorubicin uptake. For two-photon Z-stacks, this process was repeated for all of the slices of the spheroid Z-stacks to yield an average fluorescence intensity for the entirety of the measured spheroid region.

To quantify doxorubicin penetration into the spheroid, a 2-P slice 40 µm deep into the spheroid was analyzed. The spheroid region was first segmented from a transmitted light image. The average fluorescence intensity of the image at varying distances from the edge of the segmented region was then quantified. To quantify the fluorescence intensity with depth, 2.5 µm thick rings/layers of the segmented region were analyzed and quantified, using an iterative morphologic erosion operation on the segmentation region to generate each successive layer. This process is depicted in Figure S4.

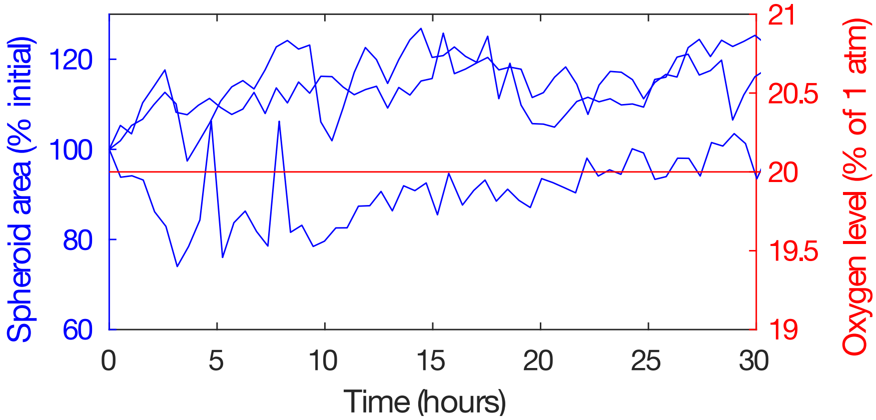

Figure S1. Segmented spheroid area vs. time for N=3 spheroids grown at constant 20% oxygen with 5% CO_2 from two separate experiments. After 30h of on-chip culture the average spheroid area was 112±16% of the initial area.

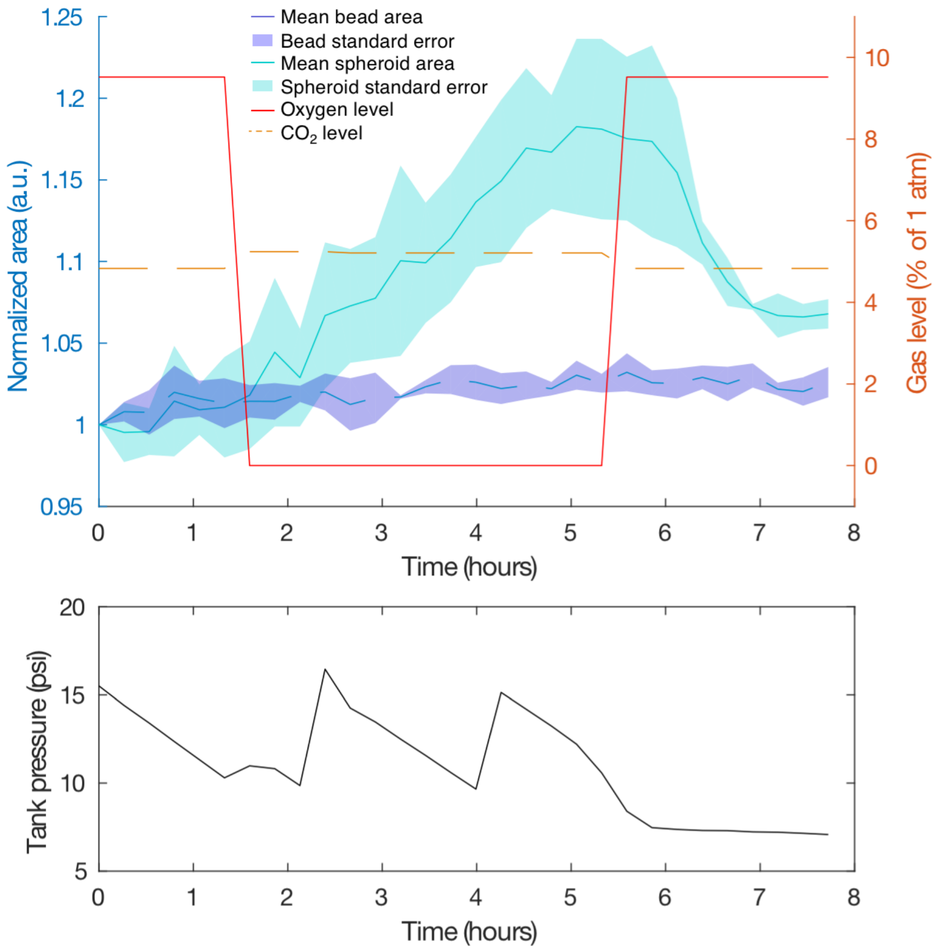

Figure S2. Comparison of the swelling behaviour of spheroids within alginate shells ('spheroid', teal) and the alginate shells themselves ('bead', blue) during exposure to a cycling oxygen profile. The mean and standard error of the normalized areas of N=3 spheroids and beads (quantified from brightfield monitoring of spheroid swelling) are plotted alongside the oxygen and CO_2 levels in the device. The pressure inside the tank during the exposure is plotted below the normalized area plot, showing poor correlation with size changes, as expected because the tank output is regulated to 5 psi. If spheroid area changes were driven by pressure from the upper layers of the microfluidic system, we would expect to see larger area changes for hydrogel beads than spheroids as the beads take up a larger volume than the spheroids within them. Taken together, it appears that oxygen level, rather than CO_2 or pressure, drives the observed spheroid swelling behaviour.

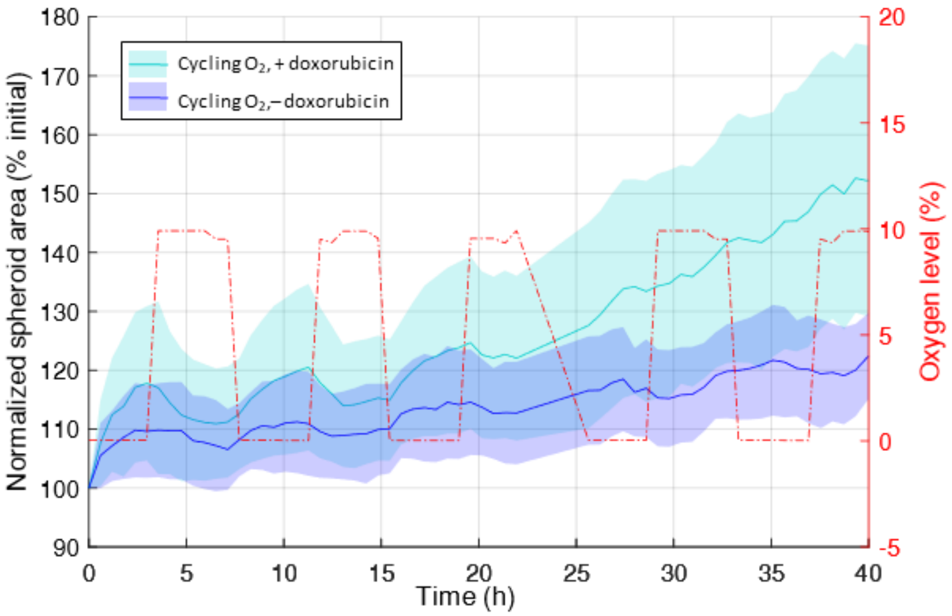

Figure S3. Comparison of the swelling behaviour of spheroids exposed to cycling oxygen profiles in the absence (blue) and presence (teal) of 10 µM doxorubicin. Size changes were quantified by brightfield microscopy. Each series depicts the mean and standard error of N=3 spheroids. The presence of doxorubicin appears to modify spheroid swelling behaviour, increasing the swelling magnitude as well as variability between spheroids.

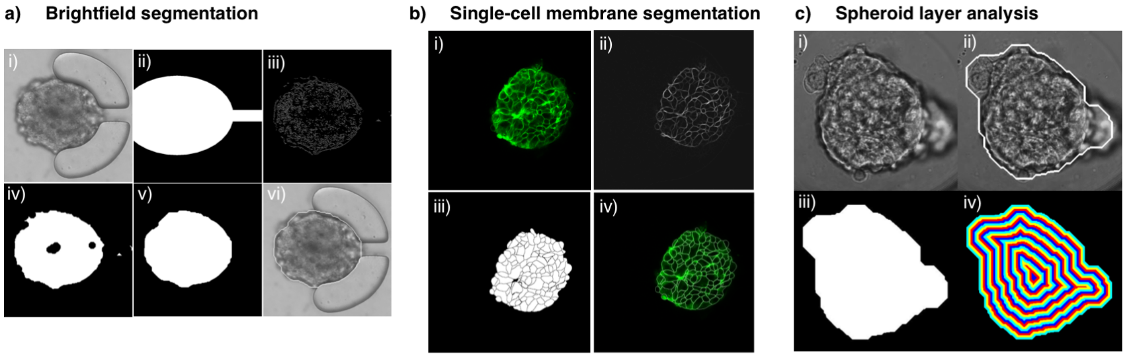

Figure S4. Image segmentation process examples. (a) segmentation of brightfield images of spheroids. (i) original image of spheroid. (ii) mask region defined to remove trap region. (iii) segmented spheroid regions (via edge detection). (iv) edges after morphologic close operation to define the spheroid region. (v) final segmentation after filling holes and morphologic open and close operations to smooth the spheroid edges. (vi) final segmentation overlaid upon the original brightfield image. (b) segmentation of single cells from two-photon fluorescence slice images of the membrane-stained spheroid. (i) original membrane stain fluorescence image. (ii) image after smoothing and ridge enhancement via the Cellsegm toolbox. (iii) final segmentation after minima finding and Watershed segmentation using the Cellsegm toolbox, as well as manual correction by blinded researchers. (iv) final segmentation overlaid upon original membrane stain image. (c) spheroid layer analysis to quantify doxorubicin penetration. (i) original transmitted light image of the spheroid. (ii) segmented transmitted light image overlaid upon the transmitted light image. (iii) segmentation region. (iv) layers of the segmentation region used to quantify the penetration. The average doxorubicin fluorescence intensity in each 2.5 µm thick ring/layer (represented as the different coloured bands in (iv)) was quantified to assess average fluorescence intensity vs. depth.

Captions for supplementary videos (provided in separate files):

Movie S1. Example time-lapse videos showing cyclic spheroid swelling over a 30h exposure to cycling hypoxia. Four example spheroids (right) were segmented (white outline on videos) and spheroid size plotted over time (left). Purple, black, blue, and green lines depict segmented spheroid areas for the four spheroids, while the red line depicts the oxygen levels supplied to the microfluidic chip. Spheroids swell at 0% oxygen and subsequently shrink at 3% and 10%. Top right spheroid was no longer within its alginate shell; all other spheroids were within alginate shells.

Movie S2. Example short time-lapse videos showing swelling of three small spheroids and non-swelling of encapsulating alginate beads during a cycling oxygen profile. The time at which each frame was acquired is annotated at the bottom right, while the gaseous oxygen level supplied to the chip during each acquisition timepoint is annotated at the top right.

Movie S3. Example time-lapse video showing two-photon monitoring of doxorubicin fluorescence in a representative spheroid exposed to cycling oxygen (between 0% and 20%) during treatment with 10 μM doxorubicin. The video depicts monitoring of a two-photon slice image approximately 50 μm deep into the spheroid. The time at which each image was acquired is reported in the bottom right, while the oxygen level at that time point is in the top right. We observe cell migration within the spheroid as well as doxorubicin uptake.

Movie S4. Example time-lapse video showing two-photon monitoring of doxorubicin fluorescence in a representative spheroid exposed to 0% oxygen (chronic severe hypoxia) during treatment with 10 μM doxorubicin. The video depicts monitoring of a two-photon slice image approximately 50 μm deep into the spheroid. The time at which each image was acquired is reported in the bottom right, while the oxygen level is in the top right. We observe cell migration within the spheroid as well as doxorubicin uptake.

Movie S5. Example time-lapse video showing two-photon monitoring of doxorubicin fluorescence in a representative spheroid exposed to 20% oxygen (in vitro normoxia, or atmospheric conditions with 5% CO_2) during treatment with 10 μM doxorubicin. The video depicts monitoring of a two-photon slice image approximately 50 μm deep into the spheroid. The time at which each image was acquired is reported in the bottom right, while the oxygen level is in the top right. We observe cell migration within the spheroid as well as doxorubicin uptake.

References

1 Hodneland, E., Kogel, T., Frei, D. M., Gerdes, H. H. & Lundervold, A. CellSegm - a MATLAB toolbox for high-throughput 3D cell segmentation. Source Code Biol Med 8, 16, doi:10.1186/1751-0473-8-16 (2013).
